# Thyroid diseases are associated with coronavirus disease 2019 infection

**DOI:** 10.3389/fendo.2022.952049

**Published:** 2022-09-02

**Authors:** Yutian Tian, Junyu Zhao, Tingting Wang, Haipeng Wang, Jinming Yao, Song Wang, Yaru Mou

**Affiliations:** ^1^ Department of Endocrinology and Metabology, The First Affiliated Hospital of Shandong First Medical University and Shandong Provincial Qianfoshan Hospital, Jinan, China; ^2^ Department of Radiology, Shandong Provincial Hospital Affiliated to Shandong First Medical University, Jinan, China; ^3^ Department of Traditional Chinese Medicine, Shandong College of Traditional Chinese Medicine, Yantai, China; ^4^ Department of Cardiology, Shandong Provincial Hospital Affiliated to Shandong First Medical University, Jinan, China

**Keywords:** thyroid diseases, hypothyroidism, coronavirus disease 2019, COVID-19, meta-analysis

## Abstract

**Background:**

In 2019, there was a global outbreak of new coronary pneumonia. Studies have found that the severity of patients with new coronary pneumonia may be related to their comorbidities. This article discusses the impact of thyroid disease on the severity of new coronary pneumonia through a meta-analysis and provides new treatment ideas for the later treatment and recovery of new coronary pneumonia.

**Methods:**

Databases including PubMed, Embase, Cochrane Library, SINOMED, China national knowledge infrastructure (CNKI), and Wanfang for coronavirus disease 2019 (COVID-19) infection and thyroid diseases were searched. Reference lists of all eligible articles and related previous review articles were handsearched. Fifty-three articles were included to conduct the meta-analysis.

**Results:**

Fifty-three articles with 12,022 COVID-19 infection patients were included in this meta-analysis. The proportion of patients with thyroid diseases in all COVID-19 infection patients fluctuates between 0% and 88.46%. Of the 53 included studies, 22 studies reported the severity of COVID-19 infection and grouped. The fixed-effects model was used to merge odds ratio (OR) values, and the pooled effect size in favor of non-severe patients is 2.62 (95% CI = 1.96–3.49, *P* < 0.0001), which means that patients with severe COVID-19 infection are more likely to have thyroid diseases. The analysis subgrouped into Asia and Europe shows that patients with COVID-19 severe infection in Asia are 3.77 times more likely to have thyroid diseases than non-severe patients (fixed-effects model: OR = 3.77, 95% CI = 2.66–5.35, *P* < 0.00001). No significant statistical heterogeneity was found by the heterogeneity analysis (chi-square = 19.85, *P* = 0.34, *I* ^2^ = 9%). Severe COVID-19 infection patients are more likely to be complicated by hypothyroidism and low T3 syndrome. The pooled ORs with fixed-effects model are 3.72 (95% CI = 1.62–8.58, *P* = 0.002) and 5.86 (95% CI = 2.79–12.33, *P* < 0.00001), respectively.

**Conclusion:**

COVID-19 infection patients with thyroid diseases are very common, and severe patients are more likely to have thyroid diseases. Asian COVID-19 infection, hypothyroidism patients, and patients with low T3 syndrome are more likely to progress to severe condition.

**Systematic Review Registration:**

https://inplasy.com, identifier INPLASY202190079.

## Introduction

Coronavirus is an enveloped single-stranded positive-stranded RNA virus of the Coronavirus family. The new 2019 novel coronavirus (2019-nCoV) is currently the seventh known coronavirus that can infect humans (1) and usually causes mild to moderate infection of the respiratory tract (1).

It has been found that patients infected with 2019-nCoV, especially those with severe disease, have high levels of immune factors such as interleukin-1B (IL-1B) and interferon gamma (IFNγ) in their blood, suggesting that cytokine concentrations are associated with the severity of the disease (2).

Evidence to date shows that having chronic diseases increases the risk of serious illnesses from coronavirus disease 2019 (COVID-19) (3). Coronaviruses are known to have direct effects on several endocrine glands, including the thyroid. In patients infected with SARS-CoV, a coronavirus related to SARS-CoV-2, autopsy revealed damage to thyroid follicles and parafollicular cells (4). Abnormalities in thyroid function have also been found to be associated with the severity of respiratory disease. Several different types of thyroid dysfunction caused by COVID-19 disease have been identified, including mild to moderate non-thyroidal disease syndrome (NTIS), severe NTIS, and subclinical hypothyroidism. All three different types of thyroid dysfunction are associated with the severity of COVID-19 prognosis (5). In one study of 61 survivors of severe acute respiratory syndrome (SARS), conducted 3 months after recovery, four (6.6%) were diagnosed to be primary hypothyroidism (6), and thyroid lesions were found in autopsies of patients who died of SARS (4). It has also been found that changes in thyroid function are often seen during the acute phase of COVID-19 (7). However, there are differences in the prevalence of thyroid disease in COVID-19 patients, which have not been comprehensively studied. Also, whether thyroid disease is associated with the severity of COVID-19 remains controversial. In addition, whether the type of thyroid disease or regional differences in patients are associated with the severity of COVID-19 remains unclear.

In this study, we performed a meta-analysis of the association between thyroid diseases and new coronary pneumonia and analyzed whether thyroid diseases were related to the severity of COVID-19. We also aimed to provide new ideas for the prognosis of new coronary pneumonia, especially the rehabilitation treatment of new coronary patients with thyroid disease.

## Materials and methods

This meta-analysis was conducted under the guidance of the Preferred Reporting Items for Systematic Reviews and Meta-Analyses (PRISMA), and it was also registered on the International Platform of Registered Systematic Review and Meta-analysis Protocols (registration number: INPLASY202190079; DOI number: 10.37766/inplasy2021.9.0079).

### Searching progress

PubMed, Embase, Cochrane Library, SINOMED, CNKI, and Wanfang were searched for studies of thyroid diseases in patients with COVID-19 infection. The literature search for this meta-analysis was restricted to published results. Databases were searched from the earliest data to 15 April 2022 with the following search terms: (“coronavirus disease 2019” OR “COVID-19”) AND (“thyroid diseases” OR “hyperthyroidism” OR “hypothyroidism” OR “thyroid function”). Reference lists of all eligible articles and related previous review articles were handsearched. Eligible studies met the following criteria: 1) published in English or Chinese language; 2) study assessed the association between COVID-19 infection and thyroid diseases (population: patients with COVID-19 infection; exposure factor: thyroid diseases; outcome: the incidence of thyroid diseases in patients with COVID-19 infection); 3) study was designed as the cohort study or case series; 4) study reported at least one result including but not limited to the number of cases, prevalence, etc.

### Study selection and data extraction

Studies were independently screened by two reviewers, and disagreements were resolved by consensus. From the eligible studies, the following data were extracted: countries and regions of inclusion of patients, study type, total number of COVID-19 infection patients, type of thyroid diseases, and number of patients with thyroid diseases.

### Methodological quality assessment

Among these 53 studies, 51 studies are retrospective cohort studies and the remaining two are case series. Newcastle–Ottawa Scale (NOS) was used to assess the methodological quality. The score of 9 is highest for NOS and shows the highest quality. Two authors scored these items independently.

### Statistical analysis

The main outcome was the number of patients with thyroid diseases in patients with COVID-19 infection. The number of patients with thyroid diseases in patients with severe disease of COVID-19 infection was also collected. Fixed-effects model was performed by computing odds ratios (ORs) and 95% CI for dichotomous variables. The *I* ^2^ was calculated as an index of heterogeneity between studies. The analyses were performed by Review Manager 5.3 (Cochrane Collaboration, United Kingdom, http://www.cochrane.org).

## Results

### Search results and characteristics of included studies

**Figure 1:**
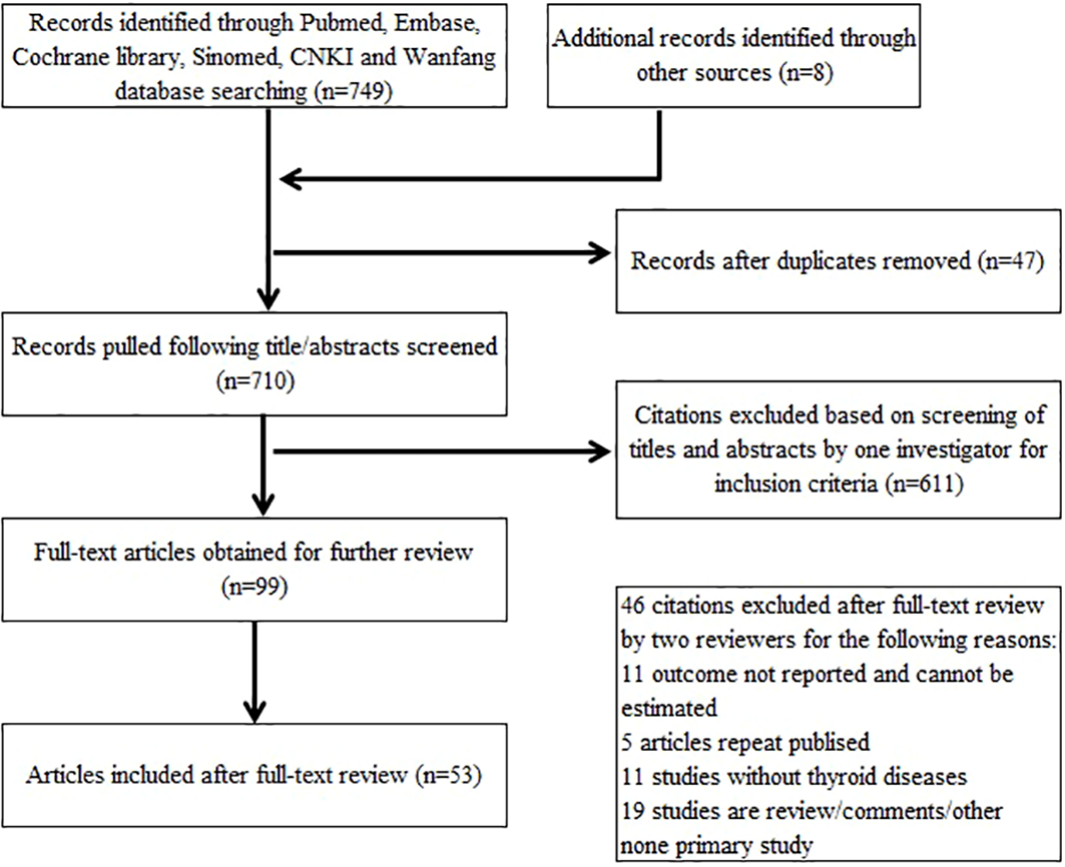
Flowchart of the systematic search process.

Our research yielded 702 articles of potentially relevant studies. Eight additional articles from the reference lists of eligible articles were also included. After screening the abstract, 99 were selected for full-text review. In total, 53 articles (3, 5, 7–57) were included in this meta-analysis. Searching progress is shown in **Figure 1**. Of the included 53 studies, 38 studies’ data are collected from Chinese COVID-19 infection patients. Italy has four articles and Saudi Arabia has three articles included. Jaipur; United States; South Korea; Kuwait; Spain; Assam, Chhattisgarh, and West Bengal; United Kingdom; and Nigeria have one article each. In total, there are 12,022 COVID-19 infection patients in all the included studies; sample size ranges from 8 to 3,707. Of these, 1,135 patients had thyroid diseases and the number was between 0 and 251. Overall, the proportion of patients with thyroid diseases in all COVID-19 infection patients fluctuates between 0% and 88.46%. Twenty-one studies reported hypothyroidism, seven studies reported hyperthyroidism, three studies reported low T3 syndrome, and the remaining 24 studies did not show the type of thyroid diseases. The detailed characteristics of the included studies are summarized in **Table 1**.

**Table 1:** Characteristics of the 53 included studies.

First author, year of publication	Country	Sample size	Number of patients with thyroid diseases	Patients with thyroid diseases/Total patients	Type of thyroid disease
Wang xuefen, 2020	Zhejiang, China	72	1	1.39%	Hypothyroidism
Zou Yilong, 2020	Hubei, China	128	2	1.56%	Hypothyroidism
Zhao Rui, 2020	Guangxi, China	28	1	3.57%	Hyperthyroidism
Xiao Miaomia, 2020	Henan, China	71	2	2.82%	Hypothyroidism
Zhao Lei, 2020	Hebei, China	21	1	4.76%	Hypothyroidism
Wang Qujue, 2020	Sichuan, China	8	1	12.50%	Hyperthyroidism
Wu Qingrong, 2020	Jiangxi, China	55	1	1.82%	Hyperthyroidism
Guo Yongfang, 2020	Hubei, China	117	22	18.80%	Low T3 syndrome
Huang Zhenghui, 2020	Hubei, China	83	3	3.61%	Hypothyroidism
Ai Xiangying, 2020	Guangdong, China	128	1	0.78%	Hypothyroidism
Li Yan, 2020	Beijing, China	9	0	0.00%	Hyperthyroidism
Liu Haichao, 2020	Hubei, China	13	2	15.38%	Hypothyroidism
Xue Hong, 2020	Jiangsu, China	15	1	6.67%	NA
Xue Hong, 2020	Jiangsu, China	31	1	3.23%	NA
Zhao Ye, 2020	Henan, China	106	2	1.89%	One hyperthyroidism and one hypothyroidism
Zhou Fawei, 2020	Hubei, China	66	1	1.52%	Hypothyroidism
Atanu Chandra, 2020	Assam, Chhattisgarh, and West Bengal	95	6	6.32%	Hypothyroidism
Runmei Zou, 2020	Hunan, China	149	41	27.52%	NA
Huang Yihui, 2020	Hubei, China	34	2	5.88%	Hypothyroidism
Sudhir Bhandari, 2020	Jaipur	21	2	9.52%	Hypothyroidism
Chen Min, 2020	Zhejiang, China	50	32	64.00%	NA
Ayman A. AI Hayek, 2020	Saudi Arabia	32	6	18.75%	NA
Andrea Lania, 2020	Italy	287	73	25.44%	31 hyperthyroidism, 2 hypothyroidism, 27 sub-hyperthyroidism and 13 sub-hypothyroidism
Sun Dan, 2020	Hubei, China	36	1	2.78%	Hypothyroidism
Maaike van Gerwen, 2020	New York, United States	3,703	251	6.78%	Hypothyroidism
Wang Zhiqiang, 2020	Hubei, China	30	1	3.33%	Hypothyroidism
Xu Ming, 2020	Henan, China	23	1	4.35%	NA
Zhang Lei, 2020	Hubei, China	386	10	2.59%	NA
Li Ruoqing, 2020	Hubei, China	193	2	1.04%	Hypothyroidism
Wan Qiu, 2020	Chongqing, China	153	4	2.61%	NA
Ilaria Muller, 2020	Italy	450	557	123.78%	15 hyperthyroidism in 204 patients
Ji yeon Lee, 2020	South Korea	694	8	1.15%	NA
Sun Ying, 2020	Beijing, China	63	3	4.76%	NA
Sulaiman Almazeedi, 2020	Kuwait	1,096	25	2.28%	Hypothyroidism
Cao Min, 2020	Shanghai, China	198	6	3.03%	NA
Liu Jinliang, 2020	Zhejiang, China	503	160	31.81%	Low T3 syndrome
Mohammed Shabrawishi, 2020	Saudi Arabia	150	9	6.00%	Hypothyroidism
Antoni Siso-Almirall, 2020	Spain	322	14	4.35%	NA
Wang Yanrong, 2020	Guangdong, China	55	2	3.64%	Hypothyroidism
Zhang Jinjin, 2020	Hubei, China	140	5	3.57%	NA
Yan Shijiao, 2020	Hubei, China	168	2	1.19%	NA
Lui DTW-1, 2021	Hong Kong, China	45	8	17.78%	NA
Lui DTW-2, 2021	Hong Kong, China	191	25	13.09%	NA
Zheng J, 2021	Hebei, China	235	51	21.70%	NA
Lui DTW-3, 2021	Hong Kong, China	122	20	16.39%	NA
Calcaterra V, 2022	Italy	26	23	88.46%	NA
Sciacchitano S, 2021	Italy	62	38	61.29%	Low T3 syndrome
Khoo B, 2021	London, United Kingdom	334	45	13.47%	NA
Alqahtani AM, 2020	Riyadh, Saudi Arabia	458	11	2.40%	NA
Lui DTW-4, 2021	Hong Kong, China	367	62	16.89%	NA
Okwor CJ, 2021	Nigeria	45	10	22.22%	NA
Zhang Y, 2021	Hunan, China	71	25	35.21%	12 euthyroid sick syndrome, 7 subclinical hypothyroidism, 4 subclinical hyperthyroidism, 2 Hypothyroidism
Wang W, 2020	Zhejiang, China	84	52	61.90%	NA

NA, None Available.

### Quality assessment of included studies

The quality of these included studies was assessed by NOS, and it was found that all of these cohort studies and case series studies get a high-quality assessment (higher than 5 scores).

### Association of the severity of COVID-19 infection with thyroid diseases

**Figure 2:**
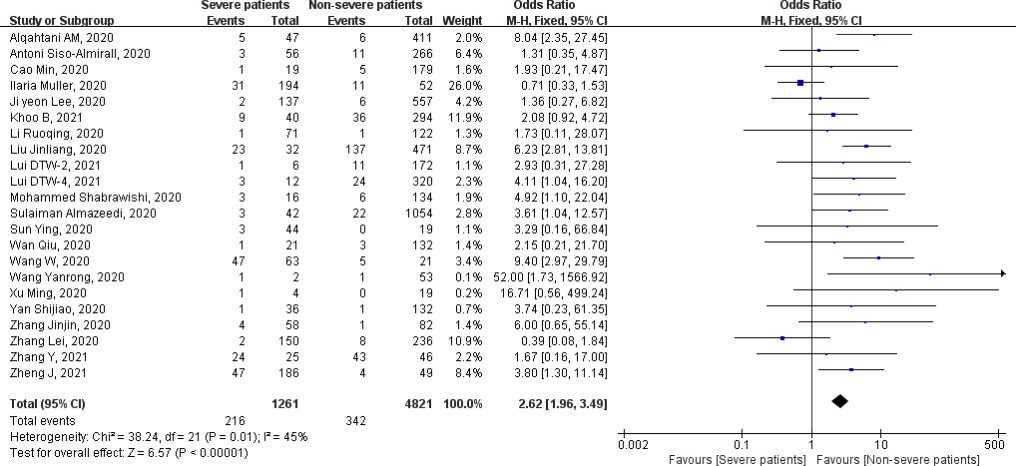
Forest plot of the association of the severity of coronavirus disease 2019 (COVID-19) infection with thyroid diseases.

**Figure 3:**
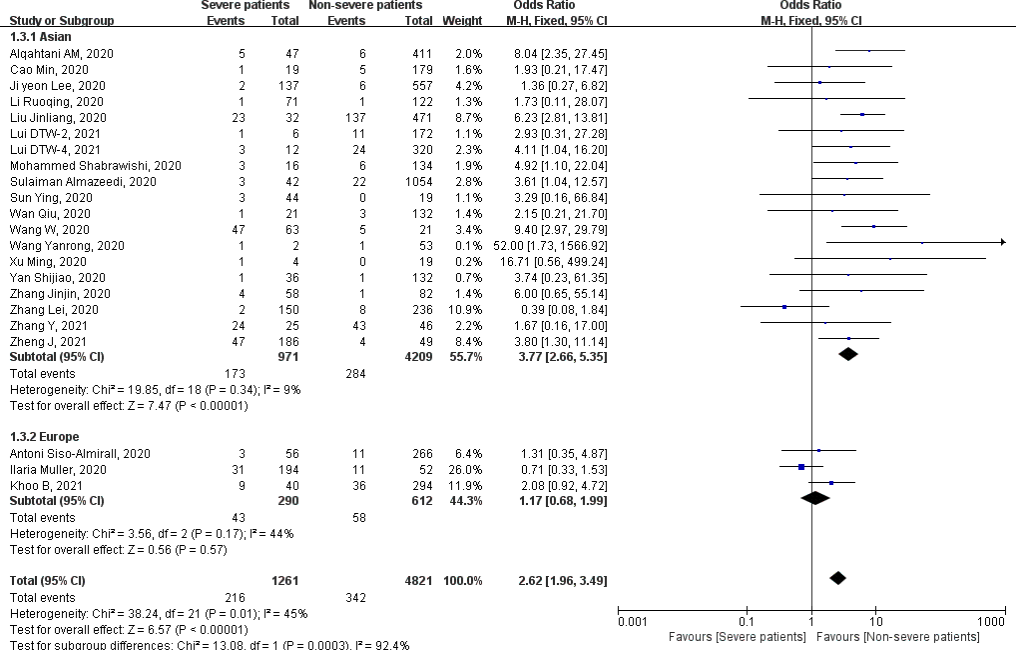
Forest plot of the association of the severity of coronavirus disease 2019 (COVID-19) infection with thyroid diseases in Asia and Europe.

**Figure 4:**
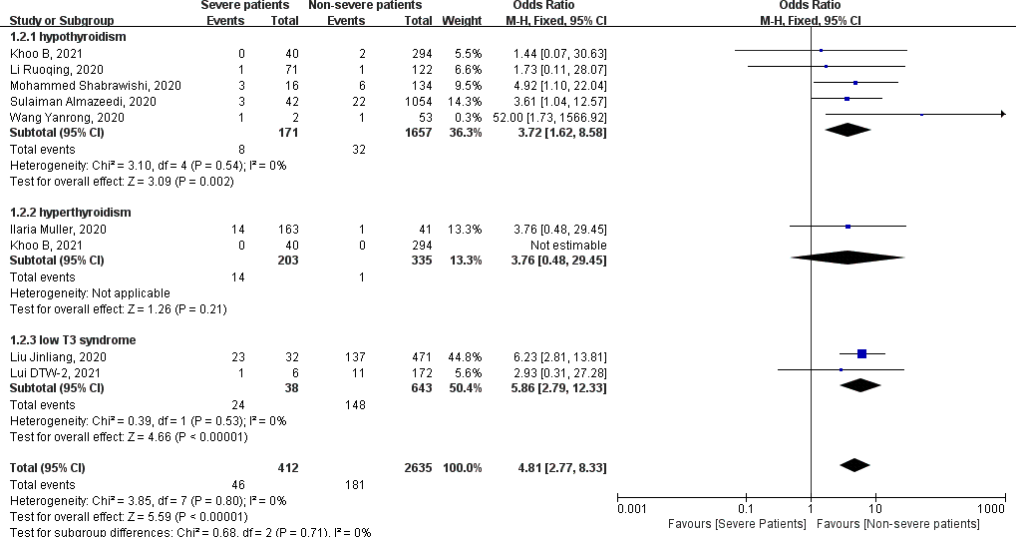
Forest plot of the association of the severity of coronavirus disease 2019 (COVID-19) infection with hypothyroidism, hyperthyroidism, and low T3 syndrome.

Of the 53 included studies, 22 studies reported the severity of COVID-19 infection and grouped. Fixed-effects model was used to merge OR values, and the pooled effect size in favor of non-severe patients is 2.62 (95% CI = 1.96–3.49, *P* < 0.0001), meaning patients with severe COVID-19 infection are more likely to have thyroid diseases. Due to the different types of thyroid diseases and different countries, there exists heterogeneity (heterogeneity test, chi-square = 38.24, *P* = 0.01, *I* ^2^ = 45%) (**Figure 2** **)**. **Figures 3**, **4** show the subgroup analysis results according to the different countries and types of thyroid diseases in patients with COVID-19 infection. Italy, Spain, and United Kingdom belong to Europe; the remaining countries including China, South Korea, Kuwait, and Saudi Arabia belong to Asia. The analysis subgrouped into Asia and Europe shows that patients with COVID-19 severe infection in Asia are 3.77 times more likely to have thyroid diseases than non-severe patients (fixed-effects model: OR = 3.77, 95% CI = 2.66–5.35, *P* < 0.00001). No significant statistical heterogeneity was found by the heterogeneity analysis (chi-square = 19.85, *P* = 0.34, *I* ^2^ = 9%). Pooled data from three studies conducted in Europe (Italy, Spain, and United Kingdom) did not show the relationship (fixed-effects model: OR = 1.17, 95% CI = 0.68–1.99, *P* = 0.57) (**Figure 3**). Subgroup analysis was further conducted by types of thyroid diseases, and the results are shown in **Figure 4**. Severe COVID-19 infection patients are more likely to be complicated by hypothyroidism and low T3 syndrome. The pooled OR with fixed-effects model in the subgroup of hypothyroidism is 3.72 (95% CI = 1.62–8.58, *P* = 0.002). No statistical heterogeneity was found by the heterogeneity analysis (chi-square = 3.10, *P* = 0.54, *I* ^2^ = 0%). Two studies analyzed the relationship of low T3 syndrome and the severity of COVID-19, and the pooled OR with fixed-effects model is 5.86 (95% CI = 2.79–12.33, *P* < 0.00001) without any heterogeneity (chi-square = 0.39, *P* = 0.53, *I* ^2^ = 0%). In conclusion, COVID-19 infection patients with thyroid diseases are very common, and severe patients are more likely to have thyroid diseases. Asian COVID-19 infection, hypothyroidism patients, and patients with low T3 syndrome are more likely to progress to severe conditions.

### Publication Bias

**Figure 5:**
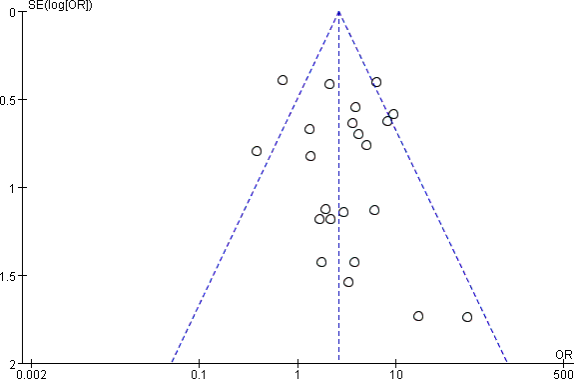
Funnel plot of publication bias.

Funnel plot was done to show the publication bias, and **Figure 5** shows a symmetrical funnel plot that indicated that no obvious publication bias existed. Moreover, the Begg’s test and Egger’s test were furtherly done. The *P* value of the Begg’s test was 0.866, and 0.434 in the Egger’s test, which means that the funnel plot is symmetric and there is no publication bias in the 22 studies selected for this meta-analysis.

## Discussion

Studies have found that patients infected with 2019-nCoV have different degrees of disease, and patients with chronic diseases may increase the risk of serious diseases. In our meta-analysis, patients with severe COVID-19 infection were found to be more likely to develop hypothyroidism and low T3 syndrome (58). This is consistent with previous studies. Studies have found that patients with lower thyroid stimulating hormone (TSH) have higher rates of fever (49) and patients with thyroid dysfunction have a poorer prognosis, and our study also found that patients with COVID-19 in Asia with hypothyroidism and low T3 syndrome were more likely to progress to severe infection.

Why does COVID-19 affect the thyroid? COVID-19 was found to affect thyroid tissue and function (59), and three different possible mechanisms have been proposed (60). The first one suggests that high expression of angiotensin converting enzyme 2 (ACE2) and transmembrane serine protease 2 (TMPRSS2) in the thyroid may contribute to SARS-CoV-2 entry. The second possible explanation is that systemic immune activation in response to SARS-CoV-2 infection may lead to thyroid damage. A third hypothesis is that the direct cytotoxic effects of the virus at the pituitary level and the indirect effects through activation of pro-inflammatory cytokine production create a “cytokine storm” that in turn induces NTIS, leading to selective transient pituitary dysregulation (61), which affects thyroid function. This is the most widely recognized reason for the mechanism.

In addition, our study found that Asian patients with COVID-19 who had hypothyroidism and low T3 syndrome were more likely to develop severe infection. So, why are patients with hypothyroidism and low T3 syndrome with neo-coronary pneumonia more likely to develop severe disease?

The invasion of COVID-19 into the body leads to an increase in IL-6, and it has been found that a decrease in free T3 concentration is associated with an increase in IL-6 (13). In addition, thyroid hormones are important in the regulation of innate immune responses (13). Therefore, excessive or insufficient levels of thyroid hormones observed in thyroid disorders would lead to an imbalance in the innate immune response. It is believed that the innate immune response is the front line of the defense system in COVID-19 infections and that a disturbed innate immune response is significantly associated with severe COVID-19 infections (62). Therefore, patients with thyroid disease may have an innate immune system disorder due to abnormal thyroid hormone levels, making patients with neo-coronary pneumonia with combined thyroid disease more likely to have exacerbations. It has been clinically shown that the clinical manifestations of patients with low T3 syndrome and severe neo-coronary pneumonia are closely correlated with various laboratory indices, which may be important indicators affecting the prognosis of patients with severe neo-coronary pneumonia. Patients with low T3 syndrome have a significantly impaired immune system, as evidenced by a significant decline in lymphocytes, which may be a key factor in the death of patients with neointimal pneumonia (8).

In conclusion, it is very common for COVID-19-infected patients to have concomitant thyroid disease, and severe COVID-19 patients are more likely to develop thyroid disease. Asian COVID-19-infected patients, patients with hypothyroidism, and patients with low T3 syndrome are more likely to develop severe disease. Whereas in the present study, the same results were not found in the European population probably due to the fact that the majority of the literature included in this paper was from Asian countries and fewer studies were from European countries, which could lead to potential bias, although no significant statistical heterogeneity was found in the heterogeneity analysis (chi-square = 19.85, *P* = 0.34, *I* ^2^ = 9%). We suggest that thyroid disease may affect the immune system, leading to a weakened ability to defend against neo-coronavirus and more likely to lead to exacerbation of the disease, but neo-coronavirus infection may also affect thyroid function by affecting immune factors, thus making this effect more pronounced. Controlling or maintaining normal thyroid function may have a positive effect on the treatment and recovery from neo-coronavirus pneumonia, and care should also be taken to maintain thyroid hormone levels during the treatment of patients with neo-coronavirus pneumonia.

## Data availability statement

The original contributions presented in the study are included in the article/supplementary material. Further inquiries can be directed to the corresponding author.

## Author contributions

YT and JZ wrote the manuscript, TW is responsible for data processing, HW is responsible for the figure drawing, JY and SW were responsible for the literature search. All authors contributed to the article and approved the submitted version.

## Funding

This study was funded by Projects of medical and health technology development program in Shandong province (grant number 2016WS0499), Shandong Provincial Natural Science Foundation of China Grants (grant number ZR2019PH025). They support the study design; the data collection, analysis and interpretation of data; the writing of the report; and the decision to submit the article for publication.

## Conflict of interest

The authors declare that the research was conducted in the absence of any commercial or financial relationships that could be construed as a potential conflict of interest.

## Publisher’s note

All claims expressed in this article are solely those of the authors and do not necessarily represent those of their affiliated organizations, or those of the publisher, the editors and the reviewers. Any product that may be evaluated in this article, or claim that may be made by its manufacturer, is not guaranteed or endorsed by the publisher.
